# *Leclercia adecarboxylata* invasive infection in a patient with Hirschsprung disease: A case report

**DOI:** 10.1016/j.amsu.2021.102927

**Published:** 2021-10-09

**Authors:** Adnane Aarab, Abderrazak Saddari, Benhamza Noussaiba, Anass Ayyad, Sahar Messaoudi, Rim Amrani, Elmostapha Benaissa, Yassine Ben Lahlou, Adil Maleb, Mostafa Elouennass

**Affiliations:** a Laboratory of Microbiology, Mohammed VI University Hospital / Faculty of Medicine and Pharmacy (University Mohammed the First), Oujda, Morocco; b Research Team “Cell Biology and Pharmacology Applied to Health Sciences”. Faculty of Medicine and Pharmacy (University Mohammed the First), Oujda, Morocco; c Department of Neonatology Intensive Care Unit, Mohammed VI University Hospital, Oujda, Morocco; d Research Laboratory for Maternal, Child and Mental Health, Faculty of Medicine and Pharmacy (University Mohammed the First), Oujda, Morocco; e Department of Bacteriology, Mohammed V Teaching Military Hospital, Rabat, Morocco; f Epidemiology and Bacterial Resistance Research Team/BIO-INOVA Centre, Faculty of Medicine and Pharmacy (University Mohammed V), Rabat, Morocco

**Keywords:** Leclercia adecarboxylata, Invasive infection, Newborn

## Abstract

**Introduction:**

*Leclercia adecarboxylata* is a ubiquitous aerobic, motile, gram-negative bacilli. The human gastro-intestinal tract is known to harbor this rarely opportunistic microorganism. We describe a rare case of invasive infection with a gastrointestinal starting point due to *L. adecarboxylata* in a patient with Hirschsprung disease.

**Case report:**

It is about a newborn female who was admitted on the 3rd day of life to the neonatal intensive care unit for intestinal obstruction. On the 9th day of life, while managing the neonatal obstruction, the patient developed febrile peaks. Cytobacteriological examination of cerebrospinal fluid, blood cultures and culture of umbilical vein catheter allowed the exclusive isolation of *Leclercia adecarboxylata*. It was producing extended spectrum beta-lactamase and was treated with intravenous imipenem*.* After favourable evolution, the patient was transferred to the pediatric surgery department. There, she was diagnosed with Hirschsprung disease.

**Discussion:**

Knowledge of the route of transmission of *L. adecarboxylata* is limited and the possible source of the infection is unclear. However, the authors describe three hypotheses of contamination of our propositus. In our patient, one or more of these routes of contamination would be possible. Indeed, bacteremia could occur as a result of a bacterial translocation across the mucosal barrier of the colon altered by Hirschsprung disease, antibiotic use and feeding practices.

**Conclusion:**

Infection with *L. adecarboxylata* revealed a wide range of infection. It has only recently been acknowledged as an emerging pathogen. Further studies of the pathogenesis and risk factors are required.

## Highlights

• *Leclercia adecarboxylata* is Gram-negative bacillus as a member of the *Enterobacteriaceae* family.
• Infection with this rarely pathogenic microorganism has been limited to a small number of case reports. Of these, only few reports implicate the gastrointestinal tract as the focus of infection.
• Bacteremia could occur as a result of a bacterial translocation across the mucosal barrier of the colon altered by Hirschsprung disease, antibiotic use and feeding practices.
• As the number of *L. adecarboxylata* infections continues to expand so does our insight into its pathogenicity and role in human clinical infections. Further studies of the pathogenesis and risk factors are required.

## 1 Introduction

*Leclercia adecarboxylata* was once thought to be a relatively uncommon pathogen [1]. Originally described by Leclerc in 1962 as *Escherichia adecarboxylata*, the development of more accurate identification methods reclassified this facultative aerobic, Gram negative bacillus as a member of the *Enterobacteriaceae* family [2].

*L. adecarboxylata is* ubiquitous and inhabits the human gastrointestinal tract [2]. Worldwide, infection with this rarely pathogenic microorganism has been limited to a small number of case reports [3,4]. Of these, only few reports implicate the gastrointestinal tract as the focus of infection [2,5–7]. In this paper we describe a case of invasive infection with a gastrointestinal starting point due to *L. adecarboxylata*, in a patient with Hirschsprung disease based on SCARE-guidelines [8]. Sharing such cases might help to elucidate risk factors for infections with this opportunistic microorganism.

## 2 Case report

It is about a newborn female born at term without incident, by caesarean section for breech presentation. The baby was admitted on the 3rd day of life (day 0) to the neonatal intensive care unit (NICU) for intestinal obstruction made of the triad: abdominal distension, postprandial bilious vomiting and failure to pass meconium. On admission (day 0), the newborn weighed 2,9 Kg for a size of 49 cm and a head circumference of 30 cm with normotensive anterior fontanel. Her pulse was 120/min, respiration rate was 40/min, temperature was 37.2 °C and oxygen saturation was 99%. On abdominal examination, we noted distended abdomen with an umbilical perimeter of 34 cm and no hepatosplenomegaly. Failure of the newborn to pass meconium resolved after a rectal tube test. Examination of the perineum, palpation of the hernia orifices and examination of the anus were normal. The abdominal X-ray, showed significant dilatation of the small intestine and the colon. The initial biological assessment was without particularity.

Enteral feeds were discontinued, intravenous fluids given and it was put on triple intravenous antibiotic therapy, made of ceftriaxone at 100 Mg/Kg/Day, gentamicin at 3 Mg/Kg/Day and amoxicillin 200 Mg/Kg/Day. On the 4th day of life (day 1), as evolution, abdomen has collapsed since the last rectal tube test and the patient was still enable to evacuate the stool. Then, it was decided to re-feed the patient and to stop nursing with monitoring of vomiting, transit and umbilical perimeter.

On the 9th days of life (day 6), while managing the neonatal obstruction, patient developed febrile peaks and laboratory tests revealed hyperleukocytosis at 13050/μl, thrombocytopenia at 6000/μl and elevation of C-reactive protein at 220,78 mg/L. Then, an infectious assessment was performed with cytobacteriological examination of cerebrospinal fluid, blood cultures, cytobacteriological examination of urine and culture of umbilical vein catheter. Technics of examination was performed in accordance with the recommendations of the Medical Microbiology Reference System (REMIC) [9]. Table 1 shows results of the different performed tests.

**Table 1:** Results of the performed tests.

Tests	Cytobacteriological examination of cerebrospinal fluid *	Blood cultures	Culture of umbilical vein catheter	Cytobacteriological examination of urine
Cytological examination results (Sysmex UF-1000i and optical microscopy)	- WBC: 82/mm3	Not performed		
	- 92% of Ly			- WBC: 26000/mL
	- 8% of PMN			- RBC: 16000/mL
Microscopic examination (Gram staning)	Gram-negative bacilli			Not performed
Culture (BD BACTEC FX400, Bloods agar plates incubation at 35 °C aerobically) **(**Blood agar plates with and without enrichment on broth vials)	Pure culture of colonies pigmented in yellow			Sterile urine culture
Presumptive identification	Aero-anaerobic, Gram-negative bacilli, oxidase negative.			Not applicable
Final identification (BD Phoenix NID Panel)	*L. adecarboxylata* (99,99% of confidence)			Not applicable

* Proteinorachy was elevated at 1,6 mg/mL and the glycorrhachia was at 0,56 mg/mL. WBC: white blood cells, RBC: red blood cells, Ly: lymphocytes, PMN: Polymorphonuclear neutrophils.

**Fig. 1:**
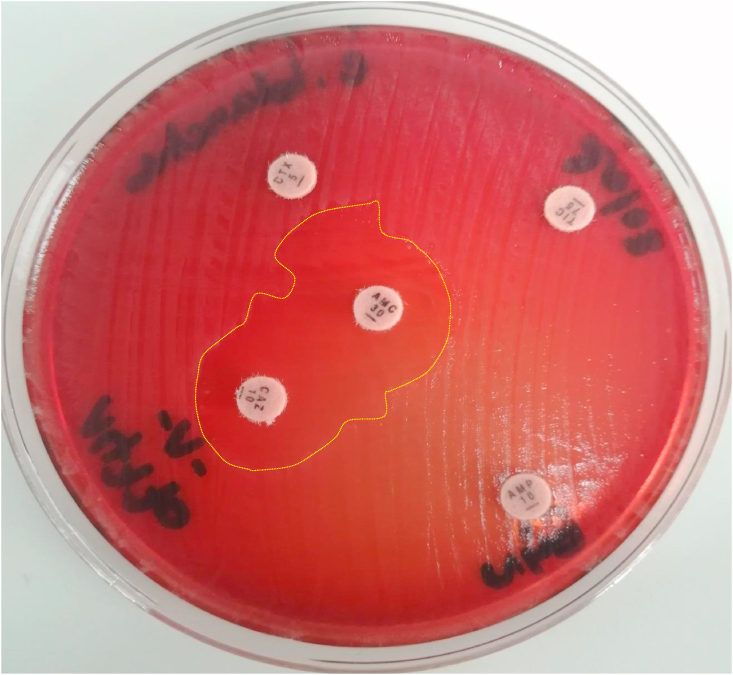
Extended-spectrum β-Lactamase (ESBL) Positive Isolates by Double Disc Synergy Test. detected by the synergistic assay between a central disc of amoxicillin + clavulanic acid (AMC) 30mm distant from the discs of cefotaxim (CTX), ceftazidime (CAZ). Dotted lines shows the dotted lines show the limits of the zone of inhibition of bacterial growth by the tested abtibiotics.

Antibiotic susceptibility testing (AST) was performed according to the European Committee on Antimicrobial Susceptibility Testing (EUCAST) [10]. The strain we isolated was producing Extended spectrum beta-lactamase (ESBL). It was resistant to penicillins without β-Lactamase inhibitors, cephalosporins (except cefoxitin) and monobactams. It was sensitive to β-Lactamase–protected penicillins, cefoxitin and carbapenems. The sensitivity of our strain to other families of antibiotics was variable. Phenotypic detection of ESBL produced by *L. adecarboxylata* is shown in Fig. 1.

On the 11th day of life (day 8), treatment was switched with imipenem at 100 Mg/Kg/Day. Repeated blood cultures taken on days 14 (day 11) and 15 (day 12) of life yielded no growth. In addition, a decrease in CRP to 5.98 Mg/L and leukocytosis at 7020/μL was noted. Then, after this favourable evolution, the patient was transferred (day 13) to the pediatric surgery department. There, she underwent a stomach surgery with multiple biopsies taken. Results of pathological anatomy confirmed that it was a Hirschsprung disease in its pancolic form.

## 3 Discussion

*L. adecarboxylata* is a Gram-negative, aero-anaerobic, mesophilic, oxidase-negative and peritrichous-flagellated bacilli [3,11]. These pigmented *Enterobacteriaceae* were described in 1962 by Leclerc. They were designated initially as ‘Enteric group 41’ or ‘*Escherichia adecarboxylata*’. However, based on nucleic acid and protein electrophoretic technics, it was separated from the genus *Escherichia*, and renamed as *L. adecarboxylata* [3,11].

*L. adecarboxylata* is distributed widely in nature and has been isolated from food, water, soil and other environmental sources. It is regarded as a normal flora in the gut of animals and gastrointestinal tract of normal humans. From clinical specimens, *L. adecarboxylata* has been isolated from blood, faeces, sputum, urine, wound pus and skin [2,3,7]. In addition, variety of diseases caused by *L. adecarboxylata* are bacteremia, sepsis, septic arthritis, diarrhea, peritonitis, gallbladder infections and meningitis. It is a common pathogen in many opportunistic *Enterobacteriaceae* species [[11], [12], [13], [14], [15], [16]].

Knowledge of the route of transmission of *L. adecarboxylata* is limited and the possible source of the infection is unclear. However, the authors describe three hypotheses of contamination of our propositus: (i) bacterial translocation across the mucosal barrier of the gastrointestinal tract, (ii) catheters or wounds entry to infect host and (iii) translocation through genitourinary tract [1,2,4,12,15]. In our patient, one or more of these routes of contamination would be possible. Indeed, bacteremia could occur as a result of a bacterial translocation across the mucosal barrier of the colon altered by Hirschsprung disease, antibiotic use and feeding practices [17]. To evaluate the association of gastrointestinal tract and bacteremia with this microorganism, we reviewed four cases reports of *L. adecarboxylata* associated with gastrointestinal tract pathology. In three of those reported cases, the patients presented only a mucosal disorder of the intestine as a possible source of infection [2,5,6]. This group of patients had not undergone any invasive interventions prior to the isolation of bacteria from their blood stream [2,5,6]. In the fourth reported case, the bacterial translocation could be the result of invasive interventions on the gastrointestinal tract suffering from mucosal alterations [7]. It can therefore be assumed that gastrointestinal pathologies causing an alteration of the mucosal barrier present a higher risk of bacteremia with this *L. adecarboxylata*. The second possible source of infection is translocation through the catheter. Indeed the contamination of catheters by the bacterial flora carried by the hands of the nursing staff is described for a set of pathogenic bacteria [4]. *L. adecarboxylata* is not an exception since it has been described by some authors in a similar situation [18]. However, in our situation, the absence of other cases of *L. adecarboxylata* infections in the services where our patient treated is against a transmission handled by the nursing staff. Concerning the urinary gateway, it is very unlikely given the absence of *L. adecarboxylata* in our patient's urinary (sterile urinary culture). In our patient, we are convinced that bacteremia occurred as a result of a bacterial translocation on a mucosal barrier altered in the colon by the pancolic form of Hirschsprung disease.

*L. adecarboxylata* has been isolated from patients with multiple germ infections. This has raised questions in some authors about the role of this bacterium in some of these infections [3,13]. In other authors, it has been suggested that this bacterium may be associated with some authentic polymicrobial infections [2]. However, a literature review of the infection with this bacterium revealed that it was the only bacterium isolated from about 62% of the infections it could cause (26 monomicrobial infections versus 16 polymicrobial infections) [14]. It therefore seems unlikely that *L. adecarboxylata* is dependent on co-infection in the case we report. Indeed, in our patient, we have not isolated any other germs associated with *L. adecarboxylata* from cerebrospinal fluid, blood or catheter cultures. Furthermore, in our opinion, the clinical improvement obtained after documented treatment on the basis of our bacteriological diagnostic, is another argument in favour of the direct responsibility of *L. adecarboxylata* in the monomicrobial infection we report. This finding underlines the rarity of the case we are reporting as it is the third case of monomicrobial *L. adecarboxylata* infection described in an immunocompetent patient [2,19]. The remaining cases reported in the literature are described in immunosuppressed patients [4].

The relative frequency of *L. adecarboxylata* in human specimens is estimated to be 2 on a scale of 0–5 [20]. However, the true frequency of infections attributed to *L. adecarboxylata* are underestimated and have been under-reported for several decades [3]. The underestimation of *L. adecarboxylata* infections results mainly from misidentification of several strains of this bacteria as *Escherichia coli,* since both species share several morphology and metabolic features [3,11,13]. Indeed, the two species could give close or even similar results on the automated system for the rapid identification of bacteria and could be easily confused in the routine laboratory if not investigated further [3,4]. In order to verify this for the *L. adecarboxylata* strain we isolated, we compared its main characters obtained on BD Phoenix PID Panel (Becton Dickinson Microbiology Systems) with the same characters described in the literature in *L. adecarboxylata* and *E. coli*. Based on the characteristics available in our identification system, Table 2 shows that all the strains we isolated (blood cultures, cerebrospinal fluid and catheter) share the same characteristics described for *L. adecarboxylata* in the literature [3,4].

**Table 2:**
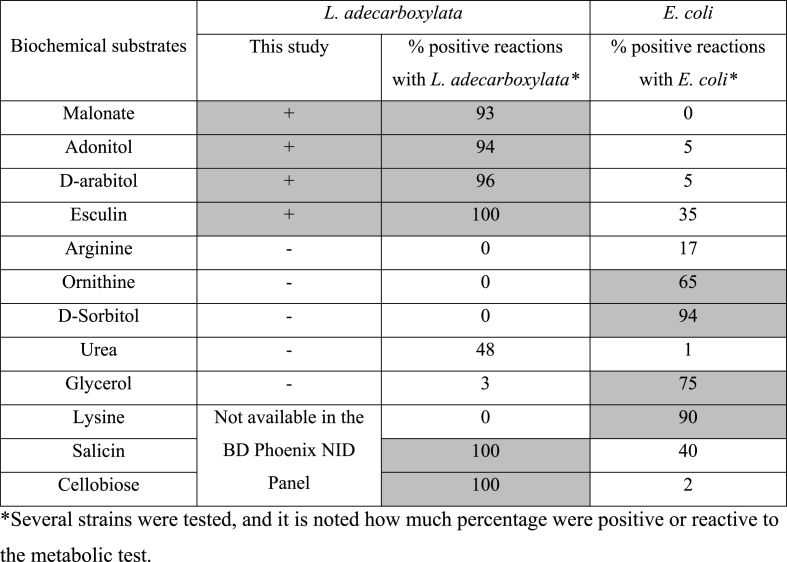
Biochemical properties of the *Leclercia adecarboxylata* strains tested.

Several reports stated that the isolates of *L. adecarboxylata* were sensitive to most antibiotics tested such as ampicillin, carbenicillin, cefaloridin, gentamicin, kanamycin, colistin, nalidixic acid, and with few exceptions, streptonycin, tetracycline, chloramphenicol and sulfadiazine [2,3,11,21] However, as the strain we have isolated, resistant strains producing ESBL were found in many studies [11,22]. Expression of this resistance phenotype by *L. adecarboxylata* would be due to a combination of two mechanisms: (i) acquiring resistance genes from other ESBL-producing bacteria and (ii) selecting the resistant strain we have isolated from the bacteria of the unbalanced intestinal flora of our proposal. The role of the broad-spectrum antibiotherapy he had received in these two mechanisms is not negligible [23,24]. Regardless of the arguments justifying the use of Amoxicillin-Ceftriaxone-Gentamicin triple therapy, producing ESBLs still complicates therapeutic management and pushes our clinician colleagues to use antibiotics of last resort such as carbapenems in the case we report.

## 4 Conclusion

Invasive infection caused by a multi-drug resistant strain of *L. adecarboxylata r*equires a rapid and an adequate management. As the number of *L. adecarboxylata* infections continues to expand so does our insight into its pathogenicity and role in human clinical infections. The factors predisposing to Infection with L. *adecarboxylata* need to be evaluated in well conducted robust studies. This will be essential to identify patients at risk to develop this infection. Patients with mucosal barrier altered should be informed of the risk of infection with *L. adecarboxylata*.

## Ethical approval

The ethical approval was not required in our institution policy. The parent of the infant gave his written consent to publish his data.

## Sources of funding

This research was not funded.

## Author contribution

All authors have made substantial contributions to all of the following: (1) the conception and design of the study (2) drafting the article and revising it critically for important intellectual content, (3) final approval of the version to be submitted.

## Registration of research studies

Not required given the case report article type.

## Guarantor

Dr Aarab Adnane,Aarabadnane@gmail.com.

## Consent

Written informed consent was obtained from the parents of the newborn for publication of this case report and accompanying images. A copy of the written consent is available for review by the Editor-in-Chief of this journal on request.

## Provenance and peer review

Not commissioned, externally peer-reviewed.

## Declaration of competing interest

None.
